# Development and function of group 2 innate lymphoid cells

**DOI:** 10.1016/j.coi.2013.02.010

**Published:** 2013-04

**Authors:** Jennifer A Walker, Andrew NJ McKenzie

**Affiliations:** MRC Laboratory of Molecular Biology, Hills Road, Cambridge CB2 0QH, UK

## Abstract

The innate lymphoid cell (ILC) family has recently expanded with the discovery of type-2 innate lymphoid cells (ILC2). These cells arise from lymphoid progenitors in the bone marrow and, under the control of the transcriptional regulators RORα and Gata3, they mature to give rise to IL-5, IL-9 and IL-13 producing ILC2. These cells are critical components of the innate immune response to parasitic worm infections and have also been implicated in the pathogenesis of asthma and allergy. Recent advances in our understanding of the molecular regulation of ILC2 development and function now present the opportunity to develop new genetic models to assess ILC2 immune function and to investigate possible therapeutic interventions.

## Highlights

► ILC2 provide an innate source of type-2 cytokines (IL-5, IL-9 and IL-13). ► IL-13 production by ILC2 is critical to anti-helminthic immunity. ► ILC2 regulate allergic airways inflammation and lung tissue homeostasis. ► ILC2 development is instructed by the transcriptional regulators, RORα and Gata3. ► Bone marrow ILC2 precursors give rise to mature ILC2 upon adoptive transfer.

**Current Opinion in Immunology** 2013, **25**:148–155

This review comes from a themed issue on **Lymphocyte development**

Edited by **Manfred Kopf** and **Hergen Spits**

For a complete overview see the Issue and the Editorial

Available online 4th April 2013

0952-7915/$ – see front matter, © 2013 Elsevier Ltd. All rights reserved.

**http://dx.doi.org/10.1016/j.coi.2013.02.010**

## Introduction

The past few years have witnessed a change in our perception of immune regulation; shifting from a view that holds T cells at the centre of immune orchestration to one that encompasses the significant contribution made by the innate immune system and particularly the newly identified cytokine-producing innate lymphoid cells (ILCs). The ILC family comprises a group of cytokine-competent cells with lymphoid morphology, which lack re-arranged antigen-specific receptors. Importantly, these cells provide a potent innate source of cytokines (that were previously primarily associated with T helper cells), and play diverse roles in lymphoid tissue formation, immunity, inflammation and tissue remodelling [1]. Consistent with their emerging roles in immune surveillance and the initiation of immune responses, ILCs are particularly prevalent at mucosal surfaces and respond to factors derived from the epithelium, which might indicate stress or microbial breach. NK cells and LTi cells represent the prototypic members of the ILC family, which has grown recently to include subsets regulated by the transcription factors RORγt (ILC3), or RORα and Gata3 (ILC2) [2,3]. ILC3 secrete predominantly IL-22 and IL-17 and have been implicated in the maintenance of intestinal barrier function and immune homeostasis [1]. ILC2 on the other hand, which form the focus of this review, provide an innate source of type-2 cytokines and are critical for the initiation of anti-helminthic and allergic immune responses. Here we summarise the functional significance of ILC2 in immunity and homeostasis and review the recent advances in our understanding of the molecular mechanisms that govern ILC2 development.

## ILC2 — a definition

ILC2 were first reported in *Rag2*^*−/−*^ mice as an IL-25-responsive, non-B non-T cell source of type-2 cytokines [4,5] and were subsequently found to provide a critical source of IL-13 in anti-helminthic immune responses [6^•^]. More recently, these cells have been characterised extensively and the names originally ascribed to them by the discovering laboratories — nuocytes [7^••^], natural helper cells (NHC) [8^••^] and innate helper 2 cells (Ih2) [9] — have been assimilated under the term ‘group 2 innate lymphoid cells’, or ILC2 [2]. ILC2 are defined by their ability to produce the type-2 signature cytokines IL-5, IL-9 and IL-13, but they also produce IL-6, IL-10, GM-CSF and small quantities of IL-4. Phenotypically, ILC2 are demarcated by the absence of conventional lineage markers (for B, T, myeloid and erythroid cells), in combination with the expression of ICOS, Sca1, IL-7Rα, CD25 and receptors for the cytokines, IL-25 (IL17BR) and IL-33 (T1/ST2). ILC2 reside in fat-associated lymphoid clusters (FALC), lymph nodes (including mesenteric (MLN) and mediastinal), spleen, liver, intestines and the airways, and originate from common lymphoid precursors (CLPs) in the bone marrow [10^••^,11]. ILC2 can be generated from CLPs *in vitro* on Delta-like ligand-expressing OP9 stromal cells in the presence of IL-7 and IL-33 [10^••^]. Whilst most of the investigations of ILC2 biology have been conducted in the mouse, similar populations have been identified in human lung and intestinal tissues and, consistent with their proposed roles in mediating type-2 immunity and inflammation, these cells are found in elevated proportions in the nasal polyps of patients with chronic rhinosinusitis [12,13^••^,14]. Human ILC2 are typically defined as lineage^−^IL-7Rα^+^NKp44^−^CD25^+^CD161^+^CRTH2^+^ [12].

## ILC2 function

### Intestinal parasitic worm clearance

Type-2 immune responses are not only required for the efficient expulsion of helminthic parasites, but are also implicated in the inflammatory processes that drive asthma and allergy. In the case of anti-helminthic immune responses, ILC2 serve as a critical early source of IL-13, which is instrumental in driving the physiological ‘weep and sweep’ processes such as goblet cell mucus secretion and contraction of intestinal smooth muscle which are required to effect worm expulsion [7^••^,8^••^,9,15,16].

*Nippostrongylus brasiliensis* is a parasite of the rat intestine that can be transmitted experimentally to mice. The parasite's life cycle includes a free-living and a parasitic intestinal phase. Immuno-competent mice clear primary infections within six to ten days with the generation of protective type-2 immunity, whilst disruption of the IL-13 pathway compromises parasite clearance [17,18]. Th2 cells were considered as an important source of type-2 cytokines in worm clearance, as SCID and *Rag2*^*−/−*^ mice lacking T and B cells cannot expel *N. brasiliensis* unless given administrations of IL-13 [19]. However, adoptive transfer of *il4/il13*^*−/−*^ T cells into *N. brasiliensis*-infected *Rag2*^*−/−*^ mice induced worm expulsion, suggesting that type-2 cytokine production was not the essential effector function of T cells in anti-helminth responses [20].

During analysis of IL-25-deficient mice, a novel cell that was induced by *N. brasiliensis* infection was identified. This cell was determined to be negative for many lineage markers, but expressed cKit, Thy1.2, and high levels of IL-13 and IL-5 [6^•^]. Although this IL-25-responsive NBNTcKit^+^IL-13^+^ cell correlated with *N. brasiliensis* expulsion, it was unclear if this cell was responsible for parasite immunity [6^•^]. The generation of IL-13eGFP reporter mice allowed identification of all the IL-13-secreting cells evoked by *N. brasiliensis* infection and showed that the majority of these cells were ILC2. Adoptive transfer of wildtype, but not IL-13-deficient, ILC2 into *N. brasiliensis*-infected *il17br/il1rl1*^*−/−*^ mice, which are severely impaired in their ability to expel worms, restored protective type-2 immunity. Indeed, transferring wildtype ILC2 into IL-13-deficient mice confirmed that IL-13 secretion from ILC2 alone was sufficient to induce parasite expulsion. Since ILC2 failed to undergo sustained expansion in helminth-infected *rag2*^*−/−*^ mice T cells appear to play an important, but as yet undefined, role in sustaining ILC2 responses. Significantly *γc*^*−/−*^*Rag2*^*−/−*^ mice infected with *N. brasiliensis* also *fail to expel N. brasiliensis* until they are reconstituted with ILC2 resulting in the restoration of goblet cell hyperplasia in the recipient mice.

### Asthma, lung inflammation and airways hyperreactivity

**Figure 1:**
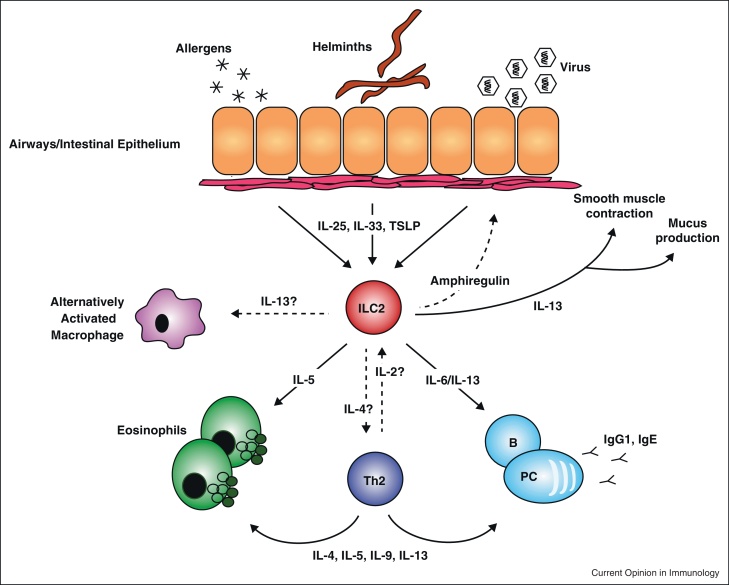
ILC2 as instigators of type-2 immunity. ILC2 play critical roles in immune responses towards helminthic parasites, viruses and allergens. In response to cytokines such as IL-25, IL-33 and TSLP, which serve as ‘distress’ signals from the epithelium, ILC produce type-2 cytokines to activate type-2 effector pathways.

Allergic asthma is a chronic inflammatory disease of the airways arising as a consequence of inappropriate immunological responses to environmental stimuli. Asthma is characterised by airways hyper-reactivity, mucus production, eosinophil and mast cell recruitment, smooth muscle contraction and airway remodelling, all of which contribute to bronchoconstriction. Recent evidence suggests that the type-2 immune response is initiated by epithelial cell-derived cytokines such as IL-25, IL-33 and TSLP (thymic stromal lymphopoietin) (Figure 1).

Notably, IL-33 or IL-25 can initiate type-2 cytokine production, accompanied by eosinophilic lung inflammation and AHR, in *Rag2*^*−/−*^ mice, independently of adaptive immunity [21,22]. The presence of IL-25-responsive and IL-33-responsive ILC2 in the naïve lung [14,22,23] is consistent with ILC2 being important in initiating type-2 responses in the airways. In fact, IL-13-producing lung ILC2 were upregulated following intranasal IL-25 or IL-33 administration, or during an ovalbumin-driven model of allergic asthma [24], and adoptive transfer of wildtype, but not IL-13-deficient, ILC2 was sufficient to restore IL-25-induced AHR to otherwise resistant IL-13-deficient mice.

The fungal aeroallergen, *Alternaria alternata* [22] has also been reported to induce ILC2 following the rapid initiation of IL-33 production in the airways, even in mice lacking B and T cells. ILC2 isolated from the lungs of wildtype mice restored the *Alternaria*-induced AHR response. Similarly, lung challenge with the glycolipid antigen, α-galactosylceramide (α-GalCer) drove IL-33 production, which appeared to be critical for the induction of IL-13-secreting ILC2. However, NKT cells also produced IL-13 in this model, and were as efficient as ILC2 in restoring AHR when adoptively transferred to *Il13*^*−/−*^ hosts.

Asthma exacerbations can be triggered by viral respiratory tract infections, and a mouse model of rhinovirus infection has revealed that viral infection correlated with elevated levels of the type-2 cytokines IL-13 and IL-4 and was associated with lung inflammation and AHR [25]. Recent reports have highlighted roles for ILC2 in experimental models of influenza virus infection [14,26]. In response influenza subtype H3N1 infection, AHR was critically dependent on IL-13 and adoptive transfer of ILC2 was capable of restoring AHR to IL-13-deficient recipients. Consistent with a role for ILC2, influenza infection-induced AHR occurred independently of the adaptive immune response, but depended on the IL-33-ST2 signalling pathway, similar to allergic asthma. These results suggest that ILC2 may be responsible for eliciting asthma exacerbation in response to influenza infection [26].

A role for ILC2 in promoting lung repair after influenza infection has also been proposed [14]. Depletion of ILC in *Rag1*^*−/−*^ mice, using an anti-CD90.2 antibody, highlighted a role for ILCs in maintaining epithelial integrity and lung function following H1N1-infected mice, and lung repair was restored by the adoptive transfer of CD90.1^+^lin^−^ST2^+^ cells. Notably, administration of recombinant IL-13 did not recapitulate this result and gene expression profiling identified a number of genes associated with tissue remodelling and wound healing including amphiregulin, a member of the epidermal growth factor family. Administration of amphiregulin was sufficient to restore lung function.

ILCs that secrete IL-22 have been identified recently in the lungs of mice during a model of experimental allergic asthma [27], and experiments using *Il22*^*−/−*^ mice demonstrated that IL-22 plays a protective role in limiting AHR and airway inflammation. Consistent with these findings, the AHR response could be exacerbated by using an anti-IL-22 antibody, or ameliorated by the administration of exogenous IL-22. Human ILC2 (Lin^−^CD127^+^ST2^+^CD161^+^CRTH2^+^) have been reported in the lung parenchyma and bronchoalveolar lavage and were also enriched in nasal polyps from patients with chronic rhinosinusitis [12,14]. These cells share a number of markers with mouse ILC2 and produce IL-13 in response to IL-25 and IL-33, but did not produce IL-17A or IL-22 and were negative for the NKp44 receptor [12].

## Transcriptional control of ILC2 development

### Losing B and T potential

ILC2, similar to other branches of the ILC family, arise from CLPs in the bone marrow and require the transcriptional inhibitor Id2 for their development [8^••^,10^••^,11]. Id2 is a member of the basic helix-loop-helix (bHLH) family of transcriptional regulators and serves to inhibit the transcription activity of E proteins, namely E12, E47, HEB and E2-2 [28]. These proteins are implicated at various stages in the differentiation of B and T cells and it is likely that suppression of the E proteins, and therefore these alternative cell fates, represents a crucial step in the ILC developmental pathway. In fact, deletion of E2A is sufficient to restore the generation of conventional NK cells in Id2-deficient mice [29].

Given the apparent importance of suppressing non-ILC developmental pathways, it is noteworthy to consider the role of Notch signalling in ILC development. Notch signalling is required to block B cell potential during T cell development [30], and subsequently provides a means to repress NK cell differentiation [31]. The timing and duration of Notch signals therefore provide critical instructions to modulate lymphoid differentiation. Recent studies suggest that Notch signalling pathways are instructive in the development of LTi, ILC3 and certain NK cell subsets [10^••^,32–35]. In the case of ILC2, Notch signalling is required for the generation of these cells from CLPs *in vitro*, although the necessity of this pathway *in vivo* has yet to be ascertained. Nevertheless, only a transient Notch signal is required in CLP cultures, consistent with a role for this pathway in suppressing alternative cell fates, rather than in instructing ILC2 differentiation *per se* [10^••^].

**Figure 2:**
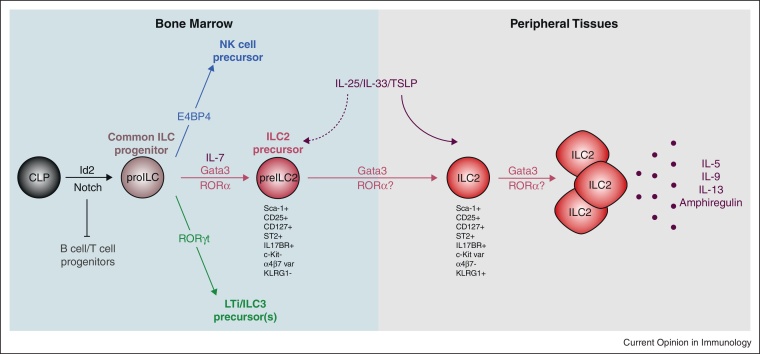
Transcriptional control of ILC2 ontogeny. ILC2 development, similar to that of other ILC family members, requires the transcriptional regulator Id2 and potentially Notch receptor signalling. Subsequent differentiation from a committed ILC progenitor (proILC) is governed by the expression of ILC lineage-specific transcription factors. ILC2 development, via an ILC2 precursor (preILC2), is directed by the transcription factors RORα and Gata3. The specific roles of these transcription factors throughout ILC2 ontogeny in the bone marrow and peripheral tissues, and in the generation of activated cytokine-producing cells, are yet to be fully elucidated.

The shared requirement for Id2 by all ILC lineages [8^••^,36–38], and potentially for Notch signalling, suggests a compelling hypothesis that invokes a common Id2^+^ precursor cell, which is directed toward particular ILC phenotypes by the expression of lineage-specific transcription factors (Figure 2) [1]. RORγt (encoded by *Rorc*) has been identified as a key regulator of ILC3 cells [38–41], whilst E4BP4 (NF-IL3) is critical to the development of NK cells [42,43]. Recently, RORα and Gata3 have been identified as key determinants of ILC2 differentiation and function, the specific roles of which will be considered in the following sections [10^••^,13^••^,44^•^,45^••^]. Consistent with this ‘common precursor’ model for ILC development, examination of LTi cell differentiation suggests that this process is governed by the sequential up-regulation of Notch, Id2 and RORγt [35], the early phase of which is accompanied by the acquisition of α4β7 expression and a loss of B cell potential [32,35,46,47].

### RORα

On the basis of gene expression data from *ex vivo* ILC2, we have recently identified RORα as a regulator of ILC2 differentiation and function [10^••^]. Using ‘*staggerer*’ mice, which carry a spontaneous deletion within the *Rora* gene [48], we have demonstrated that this transcription factor is essential for ILC2 development [10^••^], and this was corroborated recently [44^•^]. Whilst only modest reductions in ILC2 were apparent in the MLN, lung and FALC of naïve animals ([10^••^,44^•^] and JAW, unpublished), expansion of the ILC2 population in response to IL-25 injection, *N. brasiliensis* infection or intranasal papain administration was severely impaired, and characteristic type-2 immune responses failed to ensue [10^••^,44^•^]. These results suggest critical roles for RORα in at least two phases of ILC2 ontogeny; during differentiation in the bone marrow and in the response to immune challenge in peripheral tissues.

Regarding a role in ILC2 development, RORα-deficient mice lack a population of ILC2-like cells in the bone marrow (termed ‘iNH’), which display a robust capacity to generate ILC2 upon adoptive transfer [44^•^]. These cells are defined by their expression of conventional ILC2 surface molecules, such as Sca-1, IL-7Rα, CD25 and ST2, but have yet to initiate robust type-2 cytokine expression and have reduced expression of CD69, CXCR4 and CD122 compared to mature ILC2. This ILC2 precursor population appears phenotypically similar to the Gata3-dependent population identified by Hoyler *et al.*, which is discussed in the next section [45^••^].

A second potential role for RORα is in directing ILC2 proliferation and effector function. In fact, whilst ILC2-like cells can be detected in the peripheral tissues of RORα-deficient mice [10^••^,44^•^], these cells fail to expand in response to triggering cytokines. At present, it is unknown whether RORα-deficiency confers only a proliferation defect, or whether ILC2 effector function and cytokine capacity are also impaired. A challenge for the future is therefore to establish whether the ILC2-like cells that arise in RORα-deficient mice are qualitatively equivalent to their wild type counterparts.

*Rora* mRNA is broadly expressed amongst ILC populations [13^••^,45^••^] (albeit to a considerably higher degree in ILC2), and it is intriguing that the effect of *Rora* deletion is only apparent within the ILC2 subset. Indeed, the other ILC subsets are present at normal frequency in RORα-deficient mice [10^••^,44^•^]. More sophisticated mouse models in which *Rora* can be deleted in specific ILC lineages will be required to elucidate the specific roles played by RORα in ILC2 development, and to understand the mechanisms of redundancy that might exist within other ILC lineages.

The identification of RORα as a critical transcription factor in ILC2 development offers the possibility of targeting this regulator to block ILC2 generation and ameliorate allergic disease. The potential for this outcome is supported by a report that RORα-deficient mice fail to develop experimental allergic asthma [49]. ROR inhibitors have been shown to be effective in suppressing EAE, driven by RORγt-expressing Th17 cells [50], and their efficacy in targeting RORα during allergy and asthma awaits investigation.

### Gata3

Expression of Gata3 by ILC2 has been recognised by several groups [8^••^,9] and recently functional roles for this transcription factor have been identified in the ILC2 lineage. Initially, Liang *et al*. identified a Gata3-expressing ILC2 subset, which is induced during *N. brasiliensis* infection and is competent to produce IL-13 [51^•^]. Using an *Il13*-driven Cre recombinase to delete *loxP*-flanked *Gata3* alleles, these authors demonstrated that although Gata3 was dispensable for the survival of Gata3^+^IL-13^+^ ILC2, it was required for type-2 cytokine production. More recently, Hoyler *et al*. have used a tamoxifen-inducible Cre-*loxP* system, to delete *Gata3* from *Id2*-ERt2Cre expressing cells. Upon tamoxifen administration, deletion of *Gata3* resulted in the selective ablation of ILC2 cells *in vivo* and resulted in their impaired survival in culture. These studies suggest that, at least before activation and IL-13 production, Gata3 is critical for the maintenance of ILC2. Similarly, Gata3 is of functional importance for human ILC2, and partial silencing of *Gata3* resulted in reduced responsiveness of ILC2 to IL-33 and TSLP [13^••^]. Conversely, ectopic expression of Gata3 in a CRTH2^–^ILC population conferred certain attributes of the ILC2 lineage, including up-regulation of CRTH2 and expression of the receptors for IL-33 and TSLP, which in turn increased the propensity of transduced ILCs to produce type-2 cytokines [13^••^].

In addition to its expression in mature ILC2, Gata3 defines an ILC2-committed precursor population in the bone marrow, similar to the population identified by Halim *et al.* [44^•^], which can be adoptively transferred to alymphoid recipients to give rise to ILC2 in peripheral tissues [45^••^]. This lineage^–^Sca1^+^Id2^+^Gata3^+^ (or ‘LSIG’) precursor generated only ILC2 upon transfer and could not be induced to adopt alternative cell fates *in vitro*. Deletion of Gata3 resulted in ablation of the LSIG population, suggesting that it too requires Gata3 for its generation or maintenance.

Of course Gata3 is also an essential component of CD4 T cell development and the differentiation of Th2 cells [52], indicating a conserved developmental programme between Th2 and ILC2, and also the differentiation of certain NK cell subsets [53,54]. By contrast, no such function has been reported for RORα in Th2 or NK cell differentiation, though a minor role exists for RORα as a subordinate to RORγt in Th17 differentiation [55]. Clearly significant questions remain as to the molecular events that regulate Gata3 and RORα in ILC2 in comparison to T cells, and the identity of their downstream target genes.

## An ILC2 precursor (preILC2)

By virtue of their characteristic ILC2 surface marker profile (e.g. Sca1, CD25 and ST2), ILC2-like cells have been identified in the bone marrow by several groups [7^••^,44^•^,45^••^,56] though it has proven challenging to determine whether these cells truly represent precursors to the ILC2 lineage, or merely mature cells in an atypical location. Both iNH and LSIG are poor producers of cytokine and differ in their surface marker profile from ‘mature’ ILC2, for example LSIG retain α4β7 surface expression and lack the lectin-like receptor KLRG1. However, perhaps the most compelling evidence that this population might represent a true lineage committed precursor [44^•^,45^••^] is their ability to reconstitute the ILC2 compartment of alymphoid mice 20–30 times more efficiently than peripheral ILC2, without giving rise to other related lineages. With the recent rationalisation of ILC nomenclature we would suggest that these ILC2 precursors be referred to as preILC2.

## Conclusions

Thus, RORα and Gata3 are both instrumental in ILC2 development and function and, whilst neither is restricted to this lineage, the consequences to ILC development appear specific to ILC2. However, the transcriptional targets of these two factors remain to be ascertained and it will be intriguing to determine whether they overlap with those identified in T helper cell subsets. Whilst evidence exists for a potential immature precursor of ILC2, we still do not understand the extrinsic signals and transcriptional changes required for the progressive lineage restriction of CLPs to common Id2^+^ ILC progenitors, and subsequently to immature precursors of the individual ILC lineages. Elucidation of these molecular pathways presents an exciting challenge for the future.

## References and recommended reading

Papers of particular interest, published within the period of review, have been highlighted as:

• of special interest
•• of outstanding interest
